# Revisiting the Diabetes-Heart Failure Connection

**DOI:** 10.1007/s11892-018-1116-z

**Published:** 2018-10-20

**Authors:** Markus Wallner, Deborah M. Eaton, Dirk von Lewinski, Harald Sourij

**Affiliations:** 1 Division of Cardiology, Department of Internal Medicine, Medical University of Graz, Graz, Austria; 2 Cardiovascular Research Center, Lewis Katz School of Medicine, Temple University, Philadelphia, PA USA; 3 Center for Biomarker Research in Medicine, CBmed, Graz, Austria; 4 Division of Endocrinology and Diabetology, Department of Internal Medicine, Medical University of Graz, Auenbruggerplatz 15, 8036 Graz, Austria

**Keywords:** Diabetes, Antihyperglycemic drug, SGLT-2 inhibitors, Incretin-based therapy, Heart failure, Pathogenesis, Treatment

## Abstract

**Purpose of the Review:**

To summarize current clinical data investigating the link between diabetes and heart failure pathophysiology, the association of glucose control with heart failure, and the impact of current antihyperglycemic drugs on heart failure.

**Recent Findings:**

Although heart failure is one of the most prevalent outcomes occurring in real life and cardiovascular outcome trials, insufficient attention was given to this condition in diabetes research over the last decades. With both beneficial and detrimental findings for heart failure hospitalization in the health authority–mandated outcome trials for new antihyperglycemic agents, research on heart failure and its interplay with diabetes mellitus gained momentum.

**Summary:**

Diabetes mellitus and heart failure are both prevalent and intertwined conditions. While currently available heart failure therapies have a similar degree of effectiveness in patients with and without diabetes, the choice of glucose-lowering agents can substantially affect heart failure-related outcome.

## Introduction and Epidemiology

In 2014, 422 million people were affected by diabetes mellitus, and the number of patients diagnosed is estimated to increase to almost 700 million by 2040 [1, 2]. Diabetes is a global epidemic, associated with a two- to fourfold risk increase for cardiovascular diseases [3] and a two- to fivefold risk increase for developing heart failure (HF) compared to age- and risk factor-matched patients without diabetes [4]. This supports the idea of a specific intrinsic mechanism driving the distinct pathophysiological mechanism for cardiac remodeling in this population. On the one hand, advances in the management of acute coronary syndrome [5] and improvements in HF treatment have led to a significant reduction in both morbidity and mortality [6–8], but on the other hand, both improvements are thought to be responsible for an increase in prevalence of HF. Thus, HF has become one of the most prevalent cardiovascular (CV) diseases in the Western world, and its mortality exceeds most types of cancers. It is worth noting that the prevalence of HF is even higher in patients with diabetes [9, 10]. Conversely, the prevalence of diabetes is high in patients hospitalized for HF with reduced ejection fraction [11].

### Complex Interplay of HF and Diabetes

Glucose intolerance and HF are clinically and pathophysiologically interdependent, such that the worsening of one condition is frequently accompanied by worsening of the other [12]. Metabolic disorders in patients with diabetes often worsen in parallel with the progression of HF and improve after its treatment [13, 14]. Notably, it seems that HF is one of the most common complications in clinical outcome trials, especially in patients with diabetes, despite excluding patients with HF at baseline [15]. Furthermore, patients with diabetes and HF have a very poor prognosis, which is associated with high health care costs [16, 17]. There is up to a tenfold increase in mortality in patients with diabetes and HF compared to patients with diabetes without HF, which is associated with a 5-year survival rate of only 12.5% [18]. These findings underline the importance of the interplay between HF and diabetes. It is important to note that HF therapy is of similar effectiveness in patients with and without diabetes and is standardized in international guidelines [19–21]. However, the management of diabetes in HF patients seems to be less clear. While observational studies reported a significant association between lower glucose levels and less macrovascular disease, data from randomized controlled trials are generally not supportive of intensive glycemic control [22–24]. For example, in the observational analysis of the United Kingdom Prospective Diabetes Study (UKPDS) [25], an association between cardiovascular complications and glycemic control was shown, demonstrating that for every 1% decrease in circulating glycosylated hemoglobin (HbA_lc_), there was a 14% reduction in myocardial infarction (MI) events and a 16% risk reduction of HF [25].

Another study reported that during long-term follow-up of patients without CV disease, troponin T, a marker of cardiac injury, rose in parallel with HbA_lc_. This increase in troponin T was associated with future clinical outcomes (HF, death) [26]. In a recent cohort study that included over 270,000 patients with type 2 diabetes mellitus (T2DM), who were registered in the Swedish National Diabetes Register, diabetes was associated with an increased risk for hospitalization for heart failure, even if all cardiovascular risk factors were well controlled [27]. In this analysis, HbA1c was shown as a prominent factor associated with heart failure risk [27]. Large randomized controlled trials, such as VADT [22], ADVANCE [24], and ACCORD [23], which assessed the effects of intensive versus less intensive glycemic control, did not report a reduced risk of HF. The optimal level of glycemic control in patients with HF is still uncertain, with some data suggesting that tight glycemic control increases the risk of hypoglycemia, which was associated with poorer outcomes [28]. Since the Federal Drug Administration (FDA) [29] and European Medicine Agency (EMA) began mandating cardiovascular outcome trials for newly developed antihyperglycemic drugs, many cardiovascular outcome trials have been conducted. The composite primary endpoint usually selected is major adverse cardiac events (MACE), which includes CV death, non-fatal MI, and non-fatal stroke. Many of the recently conducted outcome trials have included adjudicated hospitalization for HF as a secondary endpoint. In this review, we summarize the results of the main clinical outcome trials in regard to HF [30, 31].

### Pathophysiology

Numerous factors play a role in the development of diabetes, many of which also contribute to the progression of heart failure. The underlying mechanisms involved in both diseases influence the structure, function, and metabolism of the heart. Diabetic cardiomyopathy is defined as a clinical condition of ventricular dysfunction in the absence of coronary atherosclerosis and hypertension in patients with diabetes [32]. In the early stages, diabetic cardiomyopathy is usually asymptomatic. A decrease in LV compliance, reflected by an impaired early diastolic filling and prolonged isovolumetric relaxation, is one of the earliest manifestations of diabetic cardiomyopathy [33]. Furthermore, an increase in interstitial and perivascular fibrosis has been found in the diabetic heart, which is different from the fibrosis observed following MI and with coronary artery disease or hypertension [34–36]. There is also an increase in collagen cross-linking [37]. Other structural changes include cardiac hypertrophy, which may be increased by hyperinsulinemia due to insulin resistance [38, 39]. Hyperglycemia leads to an increase in the production of mitochondrial reactive oxygen species (ROS), causing a state of oxidative stress when increased beyond the anti-oxidative capacity of the cell [40]. Oxidative stress plays a major role in downstream diabetic complications and may also be involved in gene activation and remodeling of the myocardium due to cell death mediated by ROS [39]. The diabetic heart is also characterized by an upregulation of proinflammatory cytokines, including vascular cell adhesion molecule 1; interleukins 1β, 6, and 7; monocyte chemotactic protein 1; and tumor necrosis factor-α [41, 42]. Endothelial cells, cardiomyocytes, fibroblasts, and smooth muscle cells are all impacted by this upregulation of proinflammatory cytokines and increase in ROS/oxidative stress [41]. There is also an accumulation of advanced glycation end products (AGE) in a diabetic state [43]. Dyslipidemia has been described in type 2 diabetes and is characterized by an increase in triglycerides, low HDL-C, and an increase in small-dense LDL. An increase in triglycerides causes elevated HDL catabolism, which causes the decrease in HDL-C and a shift in LDL towards the more atherogenic small-dense LDL phenotype [44]. An increase in the glycation of apolipoproteins and oxidation of LDL has also been reported [45]. Abnormalities in adipokine secretion, which includes adiponectin, leptin, apelin, and adipsin, occur in both diabetes mellitus and heart failure and may exert a multitude of downstream pathophysiological effects [46]. In vitro experiments have found insulin to have a direct positive inotropic effect via Ca^2+^-dependent and Ca^2+^-independent mechanisms using isolated human ventricular myocardium [47]. The sarcoplasmic reticulum load is increased, which increases systolic Ca^2+^ transients and the sensitivity of myofilaments [34, 47]. Other calcium abnormalities include decreased activity of sarco/endoplasmic reticulum Ca^2+^-ATPase 2a (SERCA2a) [48]. Insulin also mediates a change in the energy substrate of the heart by increasing the supply of pyruvate [49, 50]. In the diabetic heart, there is a shift in metabolic substrates, with a decrease in glucose availability [51] and an increase in fatty acid oxidation to meet energetic demands [39]. In early stages of heart failure, there is a metabolic substrate shift from fatty acids to glucose oxidation, but in later more advanced stages, insulin resistance may develop and a decrease in glucose utilization may occur [39, 52]. Studies have found insulin resistance to be an independent predictor of heart failure patients worsening prognosis [53]. Diabetic metabolic abnormalities follow the progression of heart failure, so a decline in the cardiovascular system or an improvement following treatment will be reflected metabolically [12]. However, the effects observed on heart muscle cannot be separated from those on fluid regulation. For example, insulin has been suggested to cause sodium retention in diabetes [54]. Delineating the effects of antihyperglycemic drugs on heart failure is complex and requires large-scale clinical trials.

### Insulin

HF is associated with significant changes in myocardial insulin signaling, which influences myocardial structure and function. Hence, insulin resistance in HF may contribute to adverse left ventricular remodeling and mitochondrial dysfunction [55]. In experimental studies using insulin, proinflammatory [56], vasoconstrictive [57], and anti-natriuretic [58] effects were reported, which are all likely to be undesirable in patients with HF. Furthermore, the sodium retaining effect of insulin is preserved even when other tissues are in a state of insulin resistance [59]. In some retrospective analyses of clinical trials and observational studies, insulin treatment has been consistently associated with poorer outcomes compared to non-insulin treatment strategies [60–62]. A clear limitation of observational studies is the inability to distinguish between “causality” (insulin is harmful) or a confounding effect when associations are observed, since insulin-treated patients who usually have more advanced stages of CV and renal diseases are older and have a longer history of diabetes than those treated with oral antihyperglycemic agents.

In the ORIGIN trial, which enrolled 12,537 patients with CV risk factors and impaired fasting glucose, glucose tolerance, or T2DM, subjects were randomly assigned to receive insulin or standard care. The composite co-primary endpoint (death from CV causes, non-fatal MI, non-fatal stroke, CV revascularization, or hospitalization for HF) did not differ between the insulin and standard care group (HR, 1.04; 95% CI, 0.97 to 1.11; *p* = 0.27). Hospitalization rates for HF were also similar between groups (HR, 0.90; 95% CI, 0.77–1.05) [63]. It is important to note that the ORIGIN trial also included patients with a very short history of T2DM or no diabetes diagnosis. A small randomized clinical trial of only 40 patients with T2DM and established HF compared the effects of optimized diabetes treatment with insulin to no optimization for 4 months. There was no difference in myocardial contractile reserve, oxygen consumption, and exercise capacity between the groups [64]. Taken together, there is only sufficient data for insulin glargine demonstrating a neutral effect with regard to heart failure in subjects with short diabetic history, but otherwise, the data available regarding insulin treatment in HF patients is inconclusive and requires more extensive clinical trials.

### Sulfonylureas

Sulfonylureas achieve glycemic control via an increase in insulin release by closing the ATP sensitive potassium channel on the beta cell. This mechanism of action can cause both weight gain and hypoglycemia, which can exacerbate HF.

Safety data for sulfonylureas in patients with established HF is mainly available from observational studies with varying results. Data from the Saskatchewan Health databases found sulfonylureas to increase both the mortality and hospitalization rate for HF compared to metformin (metformin vs. sulfonylureas; HR, 0.83; 95% CI, 0.70–0.99) [65]. Consistent with these findings, another retrospective analysis reported an increased mortality (metformin vs. sulfonylureas; HR, 0.54; 95% CI, 0.46–0.64) and risk for HF (metformin vs. sulfonylureas; HR, 0.76; 95% CI, 0.64–0.91) with sulfonylureas compared to metformin [66]. A Danish registry study found similar findings [67]. Conversely, Masoudi et al. found no evidence that sulfonylureas increased mortality compared to other glucose-lowering agents (HR, 0.99; 95% CI, 0.91–1.08) in a retrospective cohort study with 16,417 patients with T2DM and HF [68].

In UKPDS, newly diagnosed patients with T2DM (3867) without cardiovascular disease were randomly assigned to receive either sulfonylurea (chlorpropamide, glibenclamide, or glipizide), insulin, or conventional dietary-based treatment. HF events were comparable between the sulfonylureas (3%) and the conventionally treated group (3.3%) (HR, 0.91; 95% CI, 0.54–1.52) [69]. The sulfonylurea gliclazide was used in the ADVANCE trial for the intensive treatment group, and no significant difference in heart failure was shown between this treatment group compared to the standard control group. Since this trial was designed to compare intensive glucose control versus standard control, the effects of gliclazide and intensive glucose lowering are difficult to distinguish.

Given the lack of properly designed randomized controlled clinical trials and conflicting results from observational studies, the safety of sulfonylureas remains uncertain. The risk of hypoglycemia and weight gain, as well as the availability of other classes of antihyperglycemic drugs with proven cardiovascular safety or benefits, has reduced the use of sulfonylureas in recent years.

### Thiazolidinediones (glitazones)

Thiazolidinediones (TZDs) enhance insulin sensitivity and endothelial function while also improving the lipid profile, which may slow down the progression of atherosclerosis. The PROactive trial found that pioglitazone did not reduce the primary endpoint of all-cause mortality, non-fatal MI, non-fatal stroke, acute coronary syndrome, and revascularization (HR, 0.90; 95% CI, 0.80–1.02); while the secondary composite endpoint, including all-cause mortality, non-fatal MI, and non-fatal stroke were reduced (HR, 0.84; 95% CI, 0.72–0.98) [70]. However, TZDs cause edema and weight gain, and several clinical trials reported an increased risk of HF with TZDs. In the PROactive trial, pioglitazone significantly increased the hospitalization rate for HF compared to placebo (6% in the pioglitazone group vs. 4% in the placebo group; *p* = 0.007), without an increase in HF-related mortality [70]. Similar findings were reported in the DREAM trial, where rosiglitazone was administered to patients with impaired fasting glucose or impaired glucose tolerance without CV disease. The TOSCA.IT randomized 3028 patients with diabetes and inadequate glycemic control with metformin monotherapy to either add-on pioglitazone or a sulfonylurea. The trial was terminated early based on a futility analysis. The primary composite endpoint of all-cause death, non-fatal MI, and stroke, or urgent coronary intervention, did not differ between treatment groups (HR, 0.96; 95% CI, 0.74–1.26) [71]. Rosiglitazone was found to significantly increase the incidence of HF compared to the placebo group (rosiglitazone 0.5% vs. placebo 0.1%; HR, 7.03; 95% CI, 1.60–30.9) [72]. The RECORD trial was a multicenter, open-label trial, in which 4447 patients with T2DM on metformin or sulfonylurea monotherapy were randomly assigned to either add-on rosiglitazone or a combination of metformin and sulfonylurea (active control group). Treatment with rosiglitazone was associated with a doubled risk of HF hospitalization or HF-related death (61 vs. 29 cases; HR, 2.10; 95% CI, 1.35–3.27) [73]. Only two small randomized trials have assessed the effects of TZDs on left ventricle (LV) ejection fraction (EF) in T2DM with HF. Although there was no adverse effect on left ventricular function, treatment with TZDs was associated with an increase in BNP as a predictor for poor CV outcome [74, 75]. In 2007, the FDA gave TZDs a black-box warning for use in acute or symptomatic chronic HF patients.

### Metformin

Metformin is currently considered the primary therapeutic agent for glycemic control in T2DM based on low cost, tolerability, and the results of the UKPDS trial, which suggested improved CV outcomes [31]. However, the UKPDS trial reported very few HF events, which did not differ between metformin and conventional dietary treatment [69]. Furthermore, the results from the long-term follow-up of UKPDS did not report HF [76]. There is even less evidence regarding the effects of metformin in established HF. One randomized trial included 62 insulin-resistant HF patients treated with either metformin or placebo for 4 months. Metformin did not improve exercise capacity as assessed by peak oxygen uptake (VO_2_) [77]. As with sulfonylureas, data about the safety of metformin as a diabetes treatment in conjunction with established HF is mainly derived from observational studies. It seems that metformin use in diabetic HF patients is associated with lower mortality and morbidity compared to other antihyperglycemic agents [67, 68, 78–80]. It is worth noting that previous concerns regarding metformin causing lactic acidosis are no longer justified [81, 82] and the FDA removed heart failure as a contraindication for metformin 2006 from the label. Although metformin may be associated with better outcomes, the clear limitations of observational studies must be considered. Therefore, randomized clinical trials are required to assess whether or not metformin improves outcome in diabetic HF patients.

### DPP-4 Inhibitors

Plasma levels of dipeptidyl peptidase-4 (DPP-4) have been correlated with both human cardiac dysfunction and animal models of heart failure [83], highlighting the potential direct link between CV health and DPP family. Many cells and tissues express DPP-4 and also have exopeptidase activity against GLP-1, chemokines, and peptide hormones. DPP-4 is involved in glucose metabolism and regulation of vascular function, cell homing, and survival [84]. Other than the heart, DPP-4 inhibitors impact the vasculature, liver, immune system, kidneys, hematopoietic system, and neuroendocrine system via hormones per second messengers such as substance P, brain natriuretic peptide (BNP), release of nitric oxide, and intracellular calcium concentrations [85]. Using DPP-4 inhibitors as a therapy in different models of heart failure resulted in improvements in the severity of HF, survival, and remodeling of the ventricle [86–90]. DPP-4 inhibitor clinical outcome trials produced mixed results. Large clinical outcome trials for sitagliptin, alogliptin, and saxagliptin have been published thus far. They all reported a neutral effect on the composite primary outcome, which included non-fatal MI, non-fatal stroke, and CV mortality (plus hospitalization for unstable angina in TECOS) with hazard ratios close to 1.00. In the SAVOR-TIMI trial, which investigated saxagliptin, there was a significant increase (27%) in HF hospitalizations (3.5% for saxagliptin vs. 2.8% for placebo). The EXAMINE trial also showed a numerical yet not statistically significant increase in HF hospitalizations in the group that received alogliptin (3.1%) vs. the placebo group (2.9%). No increase in mortality was observed in either trial. In the TECOS trial, no difference in HF hospitalizations was observed between the sitagliptin vs. placebo group [91–93]. CARMELINA and CAROLINA, which are both investigating linagliptin, are in progress and the outcome data is expected in the near future. Currently, results for hospitalization for heart failure in the DPP-4 inhibitor trials are homogenous, highlighting potential differences between DPP-4 inhibitors within the same drug class. The interaction between DPP-4 inhibitors, the cardiovascular system, and more specifically cardiomyocytes has been previously established, but the direct link or mechanism connecting DPP-4 inhibitors to its effects on cardiac contractility and general function is still not fully understood. A recently published paper reported adverse off-target effects of saxagliptin on cardiac function by increasing diastolic calcium content [94]. Further mechanistic studies are necessary to develop a better understanding of the cardiovascular implications of DPP-4 inhibitors.

### SGLT-2 Inhibitors

Sodium/glucose cotransporter 2 (SGLT-2) inhibitors are an exciting new advancement in the fields of cardiology and diabetes. The EMPA-REG-OUTCOME trial [95•] reported significantly improved CV outcomes, including all-cause mortality, in patients who had received treatment with one specific anti-diabetic drug. This was the first trial to report positive CV outcomes since the European Medicines Agency (EMA) and Federal Drug Administration (FDA) [29] began requiring cardiovascular outcome trials for all new antihyperglycemic drugs. The EMPA-REG-OUTCOME trial was a randomized, double-blind, placebo-controlled trial with 7020 patients with T2DM and established cardiovascular disease. They received once-daily empagliflozin treatment (10 mg or 25 mg) or placebo treatment. There was a significant reduction (HR, 0.86; 95% CI, 0.74–0.99) of the combined primary endpoint encompassing CV death, non-fatal MI, and non-fatal stroke in T empagliflozin (pooled analysis)-treated patients during a mean follow-up of 3.1 years. These results were impacted by a decrease in CV death (HR, 0.62; 95% CI, 0.49–0.77; *p* < 0.001). Another impressive result was a 35% relative reduction in the rate of HF hospitalizations in empagliflozin-treated patients (*p* < 0.002). CANVAS and CANVAS-R [96], which are grouped together as the CANVAS program [97•], focused on canagliflozin, another SGLT-2 inhibitor. This study included 10,142 T2DM patients with a high CV risk and focused on CV safety and efficiency. Some of the results from this study were in line with the remarkable outcomes of the EMPA-REG-OUTCOME trial, while others differed. Canagliflozin significantly reduced the rate of primary outcome events, which encompasses CV death, non-fatal MI, and stroke by 14%. It also reduced HF hospitalization by 33%. Despite these positive results, canagliflozin did not significantly reduce CV or all-cause mortality. Cardiovascular outcome trials for dapagliflozin are currently in progress. The CVD-REAL (Comparative Effectiveness of Cardiovascular Outcomes in New Users of SGLT-2 Inhibitors) study, which was not a randomized controlled trial, extended the findings from both the EMPA-REG-OUTCOME trial and the CANVAS program, but this needs to be confirmed in DECLARE-TIMI 58 (NCT01730534).

The mechanism of action for how SGLT-2 inhibitors improve cardiovascular outcomes has not yet been fully described, but there are many hypotheses. Very limited data is available offering mechanistic insight, which makes this an interesting new area of investigation. Empagliflozin has been found to improve diastolic dysfunction in mouse models of diabetes, potentially through anti-fibrotic effects and increased SERCA activity [98, 99]. SGLT-2 inhibitors may also modulate energy metabolism of the myocardium, which may explain the positive CV effects [100]. Diabetic myocardium cannot oxidize fatty acids and metabolize glucose the same way a healthy heart can. SGLT-2 inhibitors may cause an increase in ketone bodies independent of diabetes, which allows for a shift in metabolic substrate utilization to fatty acids, having a beneficial impact on oxygen consumption and the work efficiency of the myocardium [100–102]. This is still an understudied area and raises many questions regarding the role of SGLT-2 inhibitors in cardiac metabolism and mechanisms of actions related to CV effects [103]. Another potential mechanism of action is that SGLT-2 inhibitors have a direct effect on the myocardium, even though there is an overall absence of cardiac SGLT-2 expression. Renal and cardiac isoforms of the sodium hydrogen exchanger (NHE) are upregulated in heart failure and diabetes, which suggests it may play a role in the relationship between the two [12, 104]. Diastolic myocardial function is positively impacted by changes in intramyocardial Na^+^ and Ca^2+^ fluxes and inhibition of NHE via SGLT-2 inhibitors [104]. Natriuresis and glucosuria have a systemic effect on hemodynamics by lowering plasma volume and blood pressure, which causes a decrease in pre-/and afterload [105, 106]. Importantly, the effects on blood pressure occurred without compensatory sympathetic activation and increase in heart rate. Additionally, a reduction in pulse pressure and arterial stiffness has been reported with SGLT-2 inhibition [107]. Another important finding is an increase in hematocrit during SGLT-2 inhibitor treatment, which is most likely mediated by the diuretic effect and an enhancement in erythropoiesis and is not associated with an increased risk for cerebral infarction. Under diabetic conditions, tubulointerstitial hypoxia induces impairments in erythropoietin production and this tubulointerstitial injury may be attenuated with SGLT-2 inhibition [108]. SGLT-2 inhibitors seem to impact the CV system independent of glucose control, which highlights their potential as a treatment option for non-diabetic HF patients [109]. The positive CV effects observed in the clinical trials speak for themselves, but determining what the mechanism of action is and if these drugs could have a beneficial impact in patients without diabetes who have a high CVD risk is very important [3]. The next group of SGLT-2 inhibitor clinical trials includes multiple phase III outcome trials in non-diabetic HF patients, with both preserved ejection fraction (HFpEF) and reduced ejection fraction (HFrEF). These trials may offer further insight into the already impressive and exciting effects of SGLT-2 inhibitors (see Table 1).

**Table 1:** Current clinical trials investigating the effects of SGLT2 inhibition in heart failure

Clinical trial	NCT number	Year of completion	Treatment	Planned number of patients	Study population	Primary outcome	Study title
EMMY	NCT03087773	2020	Empagliflozin vs. placebo	476	Patients with acute MI ± T2DM	Change in plasma concentration of NT-proBNP after 26 weeks	Impact of empagliflozin on cardiac function and biomarkers of heart failure in patients with acute myocardial infarction
EMPEROR-Reduced	NCT03057977	2020	Empagliflozin vs. placebo	2850	HFrEF (EF < 40%) ± T2DM	CV death or HF hospitalization (38 months, event-driven)	Empagliflozin outcome trial in patients with chronic heart failure with reduced ejection fraction
EMPEROR-Preserved	NCT03057951	2020	Empagliflozin vs. placebo	4126	HFpEF (EF > 40%) ± T2DM	CV death or HF hospitalization (38 months, event-driven)	Empagliflozin outcome trial in patients with chronic heart failure with preserved ejection fraction
EMPERIAL-Reduced	NCT03448419	2019	Empagliflozin vs. placebo	300	HFrEF (EF < 40%) ± T2DM	Change from baseline to week 12 in exercise capacity (6 MWT)	This study tests empagliflozin in patients with chronic heart failure with reduced ejection fraction (HFrEF). The study looks at how far patients can walk in 6 min and at their heart failure symptoms.
EMPERIAL-Preserved	NCT03448406	2019	Empagliflozin vs. placebo	300	HFpEF (EF > 40%) ± T2DM	Change from baseline to week 12 in exercise capacity (6 MWT)	This study tests empagliflozin in patients with chronic heart failure with preserved ejection fraction (HFpEF). The study looks at how far patients can walk in 6 min and at their heart failure symptoms.
EMPA-VISION	NCT03332212	2019	Empagliflozin vs. placebo	86	HFrEF (EF < 40%) and HFpEF (EF > 50%) ± T2DM	Change from baseline to week 12 in PCr/ATP ratio measured by 31P-MRS	A study that looks at the function of the heart in patients with heart failure who take empagliflozin.
Empire HF	NCT03198585	2019	Empagliflozin vs. placebo	189	HFrEF (EF < 40%) ± T2DM	Change in plasma concentration of NT-proBNP after 90 days	Empagliflozin in hear t failure patients with reduced ejection fraction
RECEDE-CHF	NCT03226457	2019	Empagliflozin vs. placebo	34	HFrEF with T2DM	Change from baseline to week 6 in urine output	SGLT2 inhibition in combination with diuretics in heart failure
REFORM	NCT02397421	2017	Dapagliflozin vs. placebo	56	HFrEF with T2DM	Changes in systolic and diastolic volumes measured by cMRI	Safety and effectiveness of SGLT2 inhibitors in patients with heart failure and diabetes
Dapa-HF	NCT03036124	2019	Dapagliflozin vs. placebo	4500	HFrEF (EF < 40%) ± T2DM	CV death or hospitalization for HF, or an urgent HF visit (time frame up to approx. 3 years)	Effect of dapagliflozin on the incidence of worsening heart failure or cardiovascular death in patients with chronic heart failure
DEFINE-HF	NCT02653482	2019	Dapagliflozin vs. placebo	250	HFrEF (EF < 40%) ± T2DM	Change in plasma concentration of NT-proBNP after 12 weeks	Dapagliflozin effect on symptoms and biomarkers in diabetic patients with heart failure
PRESERVED-HF	NCT03030235	2019	Dapagliflozin vs. placebo	320	HFpEF (EF > 45%) with T2DM or pre-diabetes	Change from baseline to week 6 and 12 in plasma concentration of NT-proBNP	Dapagliflozin effect on symptoms and biomarkers in patients HFpEF
DELIVER	NCT03619213	2021	Dapagliflozin vs. placebo	4700	HFpEF (EF > 40%)	CV death or hospitalization for HF, or an urgent HF visit (time frame up to approx. 33 months)	Dapagliflozin evaluation to improve the lives of patients with preserved ejection fraction heart failure
ERADICATE-HF	NCT03416270	2021	Ertugliflozin vs. placebo	36	HFrEF and HFpEF with T2DM	Proximal sodium reabsorption (time frame: outcome will be measured at 1 and 12 weeks)	Ertugliflozin trial in diabetes with preserved or reduced ejection fraction mechanistic evaluation in heart failure
31P-MRS	Phosphorus magnetic resonance spectroscopy						
HFrEF	Heart failure with reduced ejection fraction						
HFpEF	Heart failure with preserved ejection fraction						
6MWT	6-min walk test						
PCr/ATP	Phosphocreatine/adenosine triphosphate						
T2DM	Diabetes mellitus type 2						
cMRI	Cardiac magnetic resonance imaging						

### GLP-1 Receptor Agonists

Glucagon-like peptide-1 (GLP-1) is an incretin peptide hormone primarily synthesized by intestinal L cells [110], which is released into the circulation in response to eating, leading to glucose-dependent insulin release and suppression of glucagon. The primary active isoform is GLP-1(7-36)NH_2_, which has a half-life of 2 min. This active isoform is rapidly degraded by DPP-4 to GLP-1(9-36)NH_2_ [111], which is a GLP-1 receptor antagonist [112]. GLP-1 receptor activation also inhibits gastric and small bowel motility, reduces appetite, and ultimately leads to weight loss [113]. The GLP-1 drug class was shown to improve endothelial dysfunction, reduce infarct size post-ST segment-elevation MI, and improve cardiac output in mechanistic studies [114–117]. The beneficial effects of GLP-1 receptor agonists are thought to be the result of their direct action on the myocardium, more specifically in cardiomyocytes of the ventricle, as this is where most of these effects were reported. There have been conflicting results regarding the expression of GLP-1 receptor in cardiac tissue. Recent studies in mice and rats revealed that the GLP-1 receptor is exclusively localized in atrial cardiomyocytes [118–120].GLP-1 receptor expression in human right and left ventricular myocardium was documented by Wallner et al.; however, the expression was significantly lower compared to right atrial tissue [121]. This difference in expression may be an inherent caveat of translating basic science using animal models to humans because of the species-based differences between the two.

The first cardiovascular outcome trial for GLP-1 receptor agonists was the ELIXA trial, which studied lixisenatide. ELIXA enrolled T2DM patients who had previously suffered an acute coronary event within 180 days of screening. No significant difference was observed between the treatment and placebo group in terms of HF hospitalization or primary composite endpoint, which included cardiovascular death, MI, and stroke [122]. The SUSTAIN-6 trial tested the effect of semaglutide in T2DM patients with chronic heart failure, cardiovascular disease, chronic kidney disease, or subjects at an age ≥ 60 years with at least one cardiovascular risk factor. There was a significant reduction in the risk for the primary endpoint, which was defined as the first occurrence of non-fatal MI, non-fatal stroke, or cardiovascular disease (HR, 0.74; 95% CI, 0.85–0.95). These positive effects on composite endpoints are primarily driven by a reduction in non-fatal stroke [123]. The cardiovascular safety of liraglutide was investigated in the LEADER trial, which enrolled patients who had T2DM and 81.3% had established cardiovascular disease. The rate of the first occurrence of the primary endpoint, which included non-fatal MI, non-fatal stroke, cardiovascular disease, and all-cause mortality, was significantly reduced with liraglutide (HR, 0.87; 95% CI, 0.78–0.97). It did not cause a significant reduction in the rates of HF hospitalization, non-fatal MI, and non-fatal stroke compared to the placebo group [124]. Due to the positive results from SUSTAIN-6 and LEADER, GLP-1 receptor agonists may still ultimately improve T2DM patient’s CV outcomes. However, it seems that neither HF events nor HF hospitalization are affected by GLP-1 receptor agonists. The EXSCEL trial was another CV outcome trial that randomized 14,752 patients to either exenatide (2 mg once weekly) or placebo. The primary composite endpoint of CV death, non-fatal MI, and non-fatal stroke did not differ between exenatide and placebo (HR, 0.91; 95%CI, 0.83–1.00). There was also no difference in fatal or non-fatal MI and stroke, and hospitalization for HF and ACS [125].

The effects of liraglutide on clinical stability following hospitalization for acute HF was studied in a phase 2, double-blind, placebo-controlled trial (FIGHT trial). There was no difference in the rate of deaths (HR, 1.10; 95% CI, 0.57–2.14) or rehospitalizations for HF (HR, 1.30; 95% CI, 0.89–1.88) between liraglutide and placebo [126].

## Conclusion and Take Home Message

DM and HF are global epidemics, representing a major burden on the global health care system. A significant body of evidence indicates that the conditions are closely intertwined. HF therapies currently available have a similar degree of effectiveness in patients with and without T2DM. Tight glycemic control does not improve HF outcomes, but the choice of glucose-lowering agent can substantially affect HF-related outcome. For older glucose-lowering agents, such as insulin and sulfonylureas, there is insufficient evidence indicating the effects on HF outcomes in patients with diabetes and with established HF due to the lack of randomized controlled trials. TZDs are clearly associated with an increased risk of developing HF and worsening of pre-existing HF and should therefore be avoided in subjects at risk for HF. For saxagliptin (DDP-4 inhibitor), an increased risk for HF hospitalization has been reported. GLP-1RAs do not appear to increase the risk of developing HF, although there is uncertainty about the effects in patients with established HF. SGLT-2 inhibitors have been found to reduce the HF hospitalization in T2DM, and their safety and efficacy were confirmed in patients with diabetes with established HF. Although recent clinical trials have provided more insights regarding the efficacy and safety of glucose-lowering drugs, the available data is still insufficient for making firm evidence-based recommendations about optimal treatment of patients with diabetes and HF.
